# Multinationality mapping of public misconceptions about periodontal diseases and their public health implications

**DOI:** 10.3389/fpubh.2026.1695554

**Published:** 2026-04-20

**Authors:** Adil Abdalrahman Abalkhail, Muzammil Moin Ahmed

**Affiliations:** Department of Public Health, College of Applied Medical Sciences, Qassim University, Buraidah, Saudi Arabia

**Keywords:** misconceptions, nationalities, periodontal care, periodontal disease, public health

## Abstract

**Background:**

While much research is focused on assessing the knowledge and attitudes of periodontal patients and the general population, no study addressed the prevalence of periodontal misconceptions. Hence, the research aimed to assess misconceptions regarding periodontal diseases and their treatment among a diverse population of various nationalities.

**Methods:**

The research adopted a quantitative, descriptive, and cross-sectional approach, employing a 16-item, structured, closed-ended, validated questionnaire with perspective statements to evaluate periodontal misconceptions. The periodontal misconceptions assessment results were compared across age groups, genders, nationalities, and misconception categories.

**Results:**

Periodontal misconception assessment yielded an overall misconception ratio of 60.34% among 515 individuals of various ages, genders, nationalities, and educational backgrounds. The most prevalent misconception is that ageing leads to tooth loss (91.7%), then that periodontal diseases do not occur if oral hygiene is good (85.4%) and teeth must be brushed after every meal (85.3%). Other highly prevalent misconceptions are that periodontal treatment should not be performed during pregnancy (80.4%), and periodontal diseases can be treated solely through medication (75.7%). There was minimal misconception regarding the prevalence of periodontal diseases across all age groups, not confined to the elderly (20.7%). The mean misconception scores demonstrated no significant differences between age groups and genders. Decrease in misconceptions correlated with higher educational qualifications. Turks (16 ± 0) demonstrated the most substantial periodontal misconceptions, followed by Yemenis (13 ± 0.11), Europeans (12.13 ± 2.13), Filipinos (12.11 ± 3.69), and Bangladeshis (12.8 ± 6.62). The British (5.54 ± 2.46) exhibited the fewest misconceptions, followed by Australians (7 ± 1.41) and Indians (7.99 ± 3.85).

**Conclusion:**

The prevalence of periodontal misconceptions is high among the general public, regardless of age and gender. There exists disparity among various educational groups and nationalities, with Turks exhibiting the highest level of misconceptions, followed by Yemenis, Europeans, Filipinos, and Bangladeshis. In contrast, the British showed fewer misconceptions, followed by Australians and Indians. Mapping these misconceptions across nationalities emphasises the need to launch culturally tailored public health campaigns to improve preventive practises, reduce healthcare costs, and enhance overall population well-being.

## Introduction

Periodontal diseases are the immune-mediated breakdown of dental supporting structures that occur in consequence to the multi-microbial dysbiotic periodontal biofilm. They could manifest themselves clinically in a vast assortment of ways, such as gingival morphological alterations, gingival bleeding, oral malodour, gingival recession, pocket formation, bone loss, and loosening of the teeth (1, 2). Due to the fact that they rank second highest among dental disorders, periodontal diseases are the primary cause for concern over the state of world dental health (3). The European Federation of Periodontology’s evidence-based search, and the results of that study imply that the prevalence of mild and severe types of periodontitis are, respectively, 50 and 7.4% (4). Periodontal diseases have an impact not only on an individual’s dental and systemic health, but also on their quality of life, that can bring the feelings of low self-worth and public guilt (5, 6). It is anticipated that the expense of treating these diseases across the worldwide could equate to 54 billion dollars (7).

Periodontal diseases pose a considerable health threat due to their incidence, associations to chronic systemic diseases, financial ramifications and hampered well-being. Being associated with anxiety, masticatory dysfunction, aesthetic displeasure and social embarrassment, periodontal diseases compromise quality of life thereby public health. Considering these facts, the WHO recognises periodontal disease as a public health concern (8).

During the course of a periodontal practise, it can be clearly witnessed that patients harbour misconceptions about periodontal diseases and their treatment, during interaction with them. Periodontal misconceptions may undermine both the patients seeking periodontal care and the professionals’ capacity in rendering the periodontal treatments. In line with the findings of Pradeep et al., a significant proportion of the people who were surveyed believed that they did not require any dental treatments, which was reflected in a low utilisation of dental services (9). Patients’ concordance and want for dental care are greatly influenced by patients’ comprehension and adverse perception (10, 11). In a data collected by Gowdar et al., almost 50 % of those surveyed believed dental health misconceptions (12).

Regardless of the fact that periodontal diseases are a worry and burden for public health, there is a dearth of data concerning their treatment need, treatment demand rate, and associated factors such as misconceptions. This is supported by the analysis and suggestions provided by Alshammari et al. in their paper (13). While there is some evidence available concerning the occurrence of misconceptions about dental treatments, there is no data available for the prevalence of periodontal misconceptions. Hence, the purpose of this research was to assess the misconceptions held by the general population about periodontal diseases and their treatment.

## Methods

### Ethical considerations

The research approach is ethically and scientifically compliant with the approval of the institutional review board (# 23-60-09). Voluntary responses were obtained from the study participants.

### Study design

It is a quantitative, descriptive and cross-sectional survey. The research employed a structured closed-ended questionnaire with perspective statements to evaluate the periodontal misconceptions among the study participants. The questionnaire was validated for face, content, and construct validity. These validity were assessed through comprehensive literature review and an expert review conducted by two independent subject matter experts. The questionnaire was administered to 50 participants, constituting 10% of the overall sample size, to assess construct validity. Following the validity assessment results, modifications were made to simplify longer and unclear questions, and the 5-point Likert scale options were reduced to simple yes, no, and unsure choices to enhance clarity and ease for respondents.

### Study variables and parameters

The participants’ age, gender, nationality, and educational levels are independent variables, while perception statements about periodontal misconceptions are dependent variables. The misconception statements were grouped into three categories: misconceptions regarding oral hygiene maintenance (items 1–3), periodontal diseases (items 4–9), and periodontal disease treatment (items 10–16).

### Study population and sample size

The 500 samples of mixed gender were estimated utilising the formula delineated by Cochran et al. (14) at the power of 80%, in conjunction with Singh et al.’s guidelines for non-probability snowball sampling (15). The snowball sampling technique involved engaging various stakeholders from different countries and soliciting their participation in disseminating the questionnaire among diverse community groups, ensuring a heterogeneous representation within the sampled population. The survey’s inclusion criteria consisted of having participants over 18 years old, who provided informed consent; whereas dental professionals including dentists, dental hygienists and allied dental sciences personnel, and students, were excluded. Individuals with cognitive impairment or those who have participated in similar studies were also excluded.

### Data collection and analysis

It is an online survey with 16 misconception statements with response options of yes, no, and not sure, distributed to the general population of multiple countries via WhatsApp, emails, and Telegram. The correct responses to each question received zero point, while incorrect answers received one point. The acquired data was analysed with quantitative and descriptive statistical tests involving ANOVA and *t* test to establish misconception ratio and scores. Results of the misconceptions assessment were compared across age groups, genders, nationalities, and misconception categories. Participants belonging to constituent countries of the United Kingdom and Europe were classified as British and European, respectively, and all the participants were defined by their nationality at birth irrespective of multiple citizenships. Misconception was classified as high, average, low, and extremely low based on results ranging from 100 to 75%, 74 to 50%, 49 to 25%, and 24 to 1%, respectively. All statistical analyses were performed by an experienced senior statistician using SPSS 22.0 and R environment version 3.2.2.

## Results

A total of 515 individuals of various ages, genders, nationalities, and educational backgrounds participated in the cross-sectional survey, surpassing the estimated sample size, as outlined in Table 1. The assessment of periodontal misconceptions within the sampled population yielded an overall misconception ratio of 60.34%. The misconception ratio for each survey item is shown in Table 2. The most prevalent misconception is that ageing leads to tooth loss (91.7%), followed by the belief that periodontal diseases do not occur if oral hygiene is good (85.4%). Furthermore, it is erroneously thought that for a healthy periodontium, teeth must be brushed after every meal (85.3%). Another highly prevalent misconception is that periodontal treatment should not be performed during pregnancy (80.4%), and it is also incorrectly believed that periodontal diseases can be cured solely by taking medicines (75.7%). There was significant awareness and minimal misconception regarding the prevalence of periodontal diseases across all age groups, not confined to the elderly (20.7%). Only 34.8% of participants misconceived that children do not develop periodontal diseases, while 49.6% believed periodontal diseases resolve on their own.

**Table 1:** Demographic variables of research.

	Number of subjects	Percentage (%)
Age in years		
18–25 years	159	30.9
26–35 years	103	20.0
36–45 years	130	25.2
46–55 years	91	17.7
57–65 years	26	5.0
66 years and above	6	1.2
Gender		
Female	252	48.9
Male	263	51.1
Education		
Primary school	35	6.8
Secondary school	69	13.4
Diploma degree	45	8.7
Bachelor degree	254	49.3
Postgraduate diploma	11	2.1
Master degree	68	13.2
Doctorate/professional board	33	6.4
Nationality		
Saudi	212	41.2
Indian	77	15.0
Pakistani	20	3.9
European	18	3.5
Egyptian	18	3.5
Canadian	17	3.3
Jordanian	16	3.1
Sudanese	16	3.1
American	15	2.9
Bangladeshi	15	2.9
British	14	2.7
Filipinos	14	2.7
Yemeni	14	2.7
Australian	13	2.5
Turk	12	2.3
Chinese	12	2.3
Russian	12	2.3

**Table 2:** Performance results of misconception statements.

Misconception statement	Subjects (*n*)	Misconception ratio (%)	Misconception rate
For healthy gums, teeth should be brushed after every meal.	439	85.3	High
Gum disease will not occur if oral hygiene is good.	449	85.4	High
When oral hygiene is good, no need to get gums checked.	244	47.4	Low
Ageing by itself causes teeth to fall out.	472	91.7	High
Gum disease does Not cause serious oral health issues	163	31.7	Low
No pain means No gum disease.	275	53.4	Average
Only in elderly age can gum problems occur.	107	20.7	Very low
Just the mouth and teeth are affected by gum diseases	260	50.5	Average
Children do not get gum problems.	179	34.8	Low
Gum problems usually go away on their own.	255	49.6	Low
Gum disease can be cured solely by taking medicines.	390	75.7	High
Gum diseases treatment hurts a lot.	337	65.4	Average
Teeth become loose when they are cleaned by dentist (teeth scaling).	362	70.3	Average
Scaling (teeth cleaning) the teeth makes spaces between the teeth.	336	65.3	Average
Gum treatment affects my eye sight.	298	57.9	Average
Gum treatment should not be done during pregnancy.	413	80.4	High

**Figure 1:**
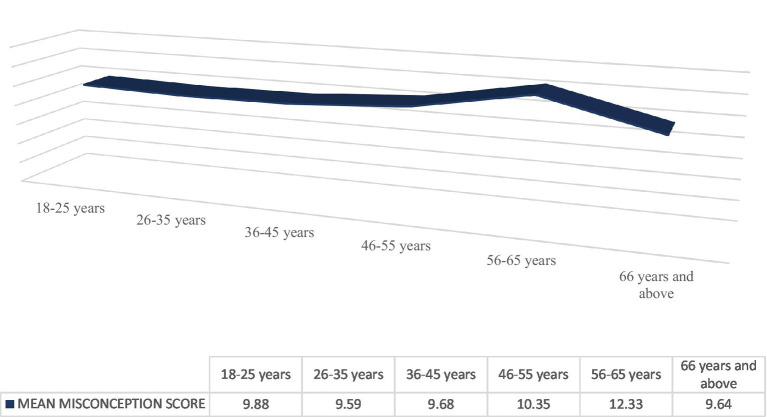
Comparison of misconception score between the age groups.

**Figure 2:**
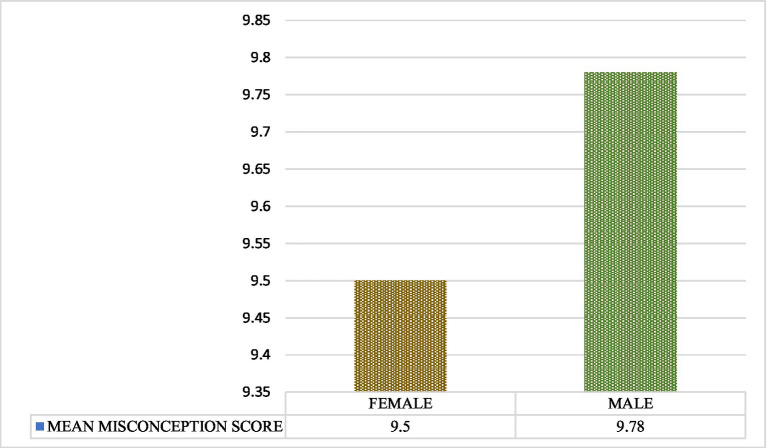
Comparison of misconception score between the genders.

The mean misconception scores demonstrated no significant differences between age groups, with an *F* value of 1.054 and a *p* value of 0.385, nor between genders, with an *F* value of 0.628 and a *p* value of 0.429, as illustrated in Figures 1, 2, respectively. Individuals with diverse educational backgrounds exhibited varying levels of misconception, with a decrease in misconceptions correlating with higher qualifications, particularly among those possessing doctoral or professional board degrees (8.3 ± 5.28), at *F* = 9.927 and *p* ≤ 0.001, as presented in Table 3.

**Table 3:** Comparison of misconception score between the education groups.

Education	Min–Max	Mean ± SD	95% confidence interval for mean	
			Lower	Upper
Primary school	6–16	13.03 ± 3.12	11.96	14.10
Secondary school	2–16	10.49 ± 4.05	9.52	11.46
Diploma	5–16	11.02 ± 3	10.12	11.92
Graduates	0–16	9.09 ± 3.6	8.65	9.54
Post graduates	0–16	8.95 ± 4.03	8.05	9.85
Doctoral/professional	0–16	8.3 ± 5.28	6.43	10.18
Total	0–16	9.64 ± 3.94	9.30	9.99

*F* value = 9.927; *p* value ≤ 0.001.

**Figure 3:**
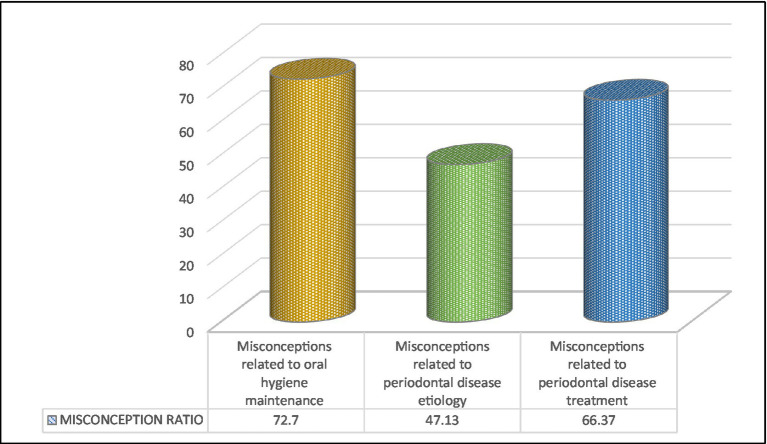
Misconception ratio for the three sections of questionnaire.

Periodontal misconception scores among participants belonging to different nationalities are highlighted in Table 3. Turks (16 ± 0) demonstrated the most substantial periodontal misconceptions, followed by Yemenis (13 ± 0.11), Europeans (12.13 ± 2.13), Filipinos (12.11 ± 3.69), and Bangladeshis (12.8 ± 6.62). The British (5.54 ± 2.46) exhibited the fewest misconceptions, followed by Australians (7 ± 1.41) and Indians (7.99 ± 3.85), while other nationalities projected a moderate degree of misconceptions, at *F* = 5.336 and *p* < 0.01 (Figure 3).

## Discussion

The study aimed to assess misconceptions regarding periodontal diseases and their treatment within the general population and employed a cross-sectional questionnaire approach that was deemed the most appropriate according to Wang et al. (16). The primary outcome of this research is a periodontal misconception prevalence of 60.34% among the general population across various nationalities. While much research is focused on assessing the knowledge and attitudes of periodontal patients and the general population, no study addresses the prevalence of misconceptions within the general population, limiting the comparison of this finding with other research. Shrivastava et al., in their short communication, raised the issue of barriers to periodontal care stemming from misconceptions regarding periodontal health (17). However, these concerns, obstacles, and their corresponding strategies hold minimal relevance without data regarding the prevalence of this misconception, and current research addressed this issue. Prabhudessai et al. identified a greater prevalence of general oral health misconceptions among pregnant patients (18), aligning with the current finding of increased misconception prevalence. The higher misconception prevalence ratio may stem from insufficient efforts by clinicians to promote awareness regarding periodontal health and its related factors. Community dental health programmes primarily provide general information on periodontal health and maintenance measures, omitting to address associated misconceptions and their potential impact on accessing periodontal care (Table 4).

**Table 4:** Comparison of misconception score in relation to nationality of subjects studied.

Nationality	Min–Max	Mean ± SD	95% confidence interval for mean	
			Lower	Upper
Turk	16–16	16 ± 0	16	16
Yemeni	10–16	13 ± 3.11	10	16
European	6–13	12.13 ± 2.13	6	13
Filipino	9–16	12.11 ± 3.69	9	16
Bangladeshi	0–16	12.8 ± 6.62	0	16
Jordanian	5–13	11.92 ± 2.39	5	13
Pakistani	5–16	11.5 ± 3.47	5	16
American	10–12	10.83 ± 0.58	10	12
Sudanese	6–16	10.4 ± 3.37	6	16
Chinese	10–14	10.33 ± 1.15	10	14
Egyptian	3–16	10.1 ± 5.43	3	16
Saudi	0–16	9.36 ± 3.83	0	16
Canadian	9–9	9 ± 0	9	9
Russian	3–16	8.33 ± 4.01	3	16
Indian	0–16	7.99 ± 3.85	0	16
Australian	6–8	7 ± 1.41	6	8
British	2–9	5.44 ± 2.46	2	9
Total	0–16	9.64 ± 3.94	0	16

*F* value = 5.336; *p* value < 0.001.

Upon comparison of the various periodontal misconceptions, the majority of participants misconceived that ageing by itself results in tooth loss, making it the most widespread among all periodontal misconceptions. Sifuentes et al. and Huange et al. reported in their epidemiological studies that edentulism and tooth loss were more prevalent among the elderly (19, 20). Nevertheless, it is worth noting that Natto et al. found that two factors associated with tooth loss in the elderly are attachment loss and a lack of dental education (21). Chan et al. and Jento et al. also indicated that tooth loss in the elderly results from dental caries, periodontal diseases, tooth wear, poor nutrition, and systemic diseases (22, 23). However, advanced age alone, in the absence of etiological factors, does not lead to tooth loss, as observed by Vargas et al. (24). The widespread misconception that ageing directly leads to tooth loss underscores the necessity for a focus on geriatric dental health among clinicians and dental students.

Another major misconception among surveyed participants is that periodontal disease does not occur with good oral hygiene. The absolute conviction that oral hygiene is the sole etiological factor of periodontal diseases contradicts the contemporary understanding that these diseases are intricate and polymicrobial, influenced by a range of risk factors including genetic, local, and systemic components (25–28). According to Weijden et al., a thorough routine for mechanical plaque removal may disrupt the periodontal pathogenic microbiota and prove beneficial in preventing periodontal diseases (29). This does not imply that excellent oral hygiene can avert the onset of periodontal diseases, as Genco et al. and Van Dyke et al. indicate that modern comprehension of periodontitis has evolved from a solely bacteria-centric model to one where the host immune-inflammatory response and individual risk factors dictate periodontal disease susceptibility and progression (30, 31). This finding of the research points out the imperative of fostering public awareness among academics and clinicians on the diverse aetiopathogenic variables linked with periodontal diseases, extending beyond mere oral hygiene considerations.

The third most prevalent misconception identified in this research is that maintaining a healthy periodontium necessitates brushing teeth after every meal. According to Attin et al., cleaning teeth once daily is sufficient enough for sustaining oral health (32). However, the literature consensus recommends brushing twice a day for optimal plaque removal (32–34). Abogazalah et al. reported that the frequency of tooth brushing varied throughout the Saudi Arabian population, with just a small proportion adhering to the recommended guidelines (35). The misconception regarding brushing frequency stems from public unawareness and should be addressed swiftly through community oral health programmes and the introduction of dentist-assisted periodontal care strategies.

The current study found that a large majority of research participants considered that periodontal treatment should not be undertaken during pregnancy. This misconception corresponds with the conclusion drawn by Javali et al. in their study, which states that misconceptions about pregnancy and oral health act as barriers to accessing dental treatment (36). This misconception may arise from the insufficient education provided to women by gynaecologists, medical practitioners, and dental professionals, which may be attributed to a lack of knowledge and awareness among these professionals, as demonstrated by Tarannum et al. in their study of health professionals (37). Another highly prevalent misconception found in this research is that the periodontal diseases may be managed exclusively with medicine. This presumption fundamentally misapprehends the polymicrobial characteristics and antimicrobial resistance of periodontal biofilm that eventually result in periodontal diseases (38, 39). Despite the prevalence of multiple periodontal misconceptions, there were few misconceptions about the occurrence of periodontal diseases across all age groups. This prevalent notion may have arisen from rising observations of the ubiquitous nature of periodontal diseases (40).

The current research found that these misconceptions were not confined to any specific demographic stratum but were, instead, equally widespread among both males and females and across all age groups. Mona Rajeh, and Al Harthi in their cross-sectional studies, reported a finding that contradicts the results of this research, indicating that females exhibited greater oral health comprehension compared to males (41, 42). According to the 10-year incidence study of Solveig Fure, older adults often have a more “fatalistic” attitude than the younger adults (43). Yet, such observations were not found in this study about periodontal misconceptions, indicating a lack of awareness of periodontal health and diseases, as well as the widespread occurrence of periodontal misconceptions regardless of age or gender. However, there were considerable disparities in the prevalence of periodontal misconceptions among individuals with various educational backgrounds. According to Baig et al., Higher levels of education correlated with correct beliefs, while advanced age correlated with misconceptions (44). The disparity among the individuals with varied educational levels can be traced to the concept of health literacy; thus, higher educational attainment is substantially associated with increased functional health literacy (45, 46).

The most notable and intriguing aspect of this research is the comparison of periodontal misconceptions across various nationalities. The comparative investigation revealed significant misconception disparities among different nationalities. Turks had the most profound periodontal misunderstandings, with Yemenis, Europeans, Filipinos, and Bangladeshis following closely behind. The British displayed the fewest misconceptions, followed by Australians and Indians, while other nationalities indicated a moderate level of misconceptions. These variations can be ascribed to cultural practises shaped by nationality, which in turn influence an individual’s perception of health and the accessibility of health services (47, 48). This research outcome also suggests that misconceptions about periodontal health are not randomly distributed; instead, they are profoundly influenced by a complex confluence of socioeconomic, educational, and healthcare-system factors. The better outcomes for individuals from the UK and Australia may be due to their long-standing, established, and accessible healthcare systems that focus on preventing disease through the integration of oral health strategies (49, 50). Fewer periodontal misconceptions among Indian participants, positioning them similarly to Australians and slightly behind the British, are indicative of the swift expansion and high prominence of the dental profession in urban India, heightened media attention on oral health and aesthetics, and the impact of a substantial English-speaking demographic with enhanced access to global health information online (51).

The research outcomes uncovered widespread misconceptions among the general population regarding multiple aspects of periodontics and brought out several socioeconomic variables linked to these misconceptions. The relevance of this research resides in its capacity to bridge the research gap and its merits for dental healthcare professionals, the general public, the scientific community, and public health policymakers. Periodontal diseases are one of the most prevalent diseases that impact not only oral health but also general well-being. Despite this, the demand for periodontal care among patients is not as high as for other restorative and prosthetic care, which can be ascribed to widespread misconceptions among the general public. This research will draw the attention of dental clinicians to emphasise the necessity of educating their patients about potential periodontal misconceptions and to motivate them to seek better periodontal care, thereby reducing the burden on dental healthcare. Additionally, the scientific community will be able to leverage the outcomes of this investigation as crucial baseline data for subsequent research aimed at measuring future knowledge changes and, eventually, health impacts.

The merits of this research reside in its novelty, rigorously devised and executed design that promotes reproducibility, distinctive findings pertaining to different nationalities, and its applicability to various healthcare stakeholders. The limitations of this research stem from the smaller sample size, which inadequately represents many nationalities, including those from Asian and African countries, due to the non-probability nature of the sampling technique. The cross-sectional design of this research precludes the potential to monitor changes in periodontal misconceptions over time or in response to educational interventions. While measures are implemented to mitigate the methodological bias associated with snowball sampling, including the homogeneity of the sampling method, the other limitations inherent to this technique cannot be ruled out. Nevertheless, the potential positive impact of this research outweigh the limitations and provide broad scope for further research.

### Public health implications

The research will raise public awareness of prevalent misconceptions about periodontal diseases and enable the public to understand how to prevent and obtain their treatment correctly. The research findings will support a transformation in public health education, emphasising the critical need to develop focused public health initiatives that correct periodontal misconceptions. One of the most significant implications that could come out of this research is the possibility of encouraging the inclusion of periodontal health education in general healthcare policy. Periodontal misconceptions are not consistently distributed throughout nationalities, resulting in an unequal distribution of periodontal burden. By comprehending the specific periodontal misconceptions held by nationalities, public health authorities may develop equity-focused initiatives that involve conscious allocation of resources through channels accessible to marginalised populations.

## Conclusion

The prevalence of misconceptions regarding periodontal diseases and their prevention and treatment is notably high among general public, showing no significant differences based on age or gender. However, there exists a significant distinction among various educational groups and nationalities, with Turks exhibiting the highest level of misconceptions, followed by Yemenis, Europeans, Filipinos, and Bangladeshis. In contrast, the British showed fewer misconceptions, followed by Australians and Indians.

## Data Availability

The original contributions presented in the study are included in the article/supplementary material, further inquiries can be directed to the corresponding author.
